# seqCNA: an R package for DNA copy number analysis in cancer using high-throughput sequencing

**DOI:** 10.1186/1471-2164-15-178

**Published:** 2014-03-05

**Authors:** David Mosen-Ansorena, Naiara Telleria, Silvia Veganzones, Virginia De la Orden, Maria Luisa Maestro, Ana M Aransay

**Affiliations:** CIC bioGUNE & CIBERehd, Technologic Park of Bizkaia, Building 502, 48160 Derio, Spain; Clinical Analyses Service at the San Carlos Clinical Hospital, Martin Lagos, 28040 Madrid, Spain; Dominion Pharmakine S.L, Technologic Park of Bizkaia, Building 801, 48160 Derio, Spain

**Keywords:** High-throughput sequencing, Cancer, Copy number, R, Bioconductor

## Abstract

**Background:**

Deviations in the amount of genomic content that arise during tumorigenesis, called copy number alterations, are structural rearrangements that can critically affect gene expression patterns. Additionally, copy number alteration profiles allow insight into cancer discrimination, progression and complexity. On data obtained from high-throughput sequencing, improving quality through GC bias correction and keeping false positives to a minimum help build reliable copy number alteration profiles.

**Results:**

We introduce *seqCNA*, a parallelized R package for an integral copy number analysis of high-throughput sequencing cancer data. The package includes novel methodology on (i) filtering, reducing false positives, and (ii) GC content correction, improving copy number profile quality, especially under great read coverage and high correlation between GC content and copy number. Adequate analysis steps are automatically chosen based on availability of paired-end mapping, matched normal samples and genome annotation.

**Conclusions:**

*seqCNA*, available through Bioconductor, provides accurate copy number predictions in tumoural data, thanks to the extensive filtering and better GC bias correction, while providing an integrated and parallelized workflow.

**Electronic supplementary material:**

The online version of this article (doi:10.1186/1471-2164-15-178) contains supplementary material, which is available to authorized users.

## Background

Genomic structural rearrangements are a hallmark of cancer. Among them, deviations in the amount of genomic content that arise during tumorigenesis, called copy number alterations (CNAs), can critically affect gene expression patterns [1, 2]. A priori, expression could be expected to correlate with gene dosage. However, although a certain global correlation exists, individually, it has not been observed to be linear and, in some cases, it can even be inverse [3]. This is due to a range of dosage regulation mechanisms [4] that confer the cell with robustness to the presence of CNAs. Still, greater expression variability arises from such regulation [5] and, if large amounts of DNA are affected by CNAs, cell control cannot be kept [6]. Furthermore, post-transcriptional and post-translational modifications, folding stability and gene-protein and protein-protein interactions greatly mask the effect of copy number changes, but correlation between gene dosage and protein expression has been found to be greater in the case of oncogenes [7]. Indeed, the importance of CNAs in cancer is demonstrated by the existence of CNA patterns that allow to differentiate between cancer types [2, 8] and to analyze cancer progression and complexity [9].

High-throughput technologies, including array comparative genomic hybridization (aCGH), single nucleotide polymorphism (SNP) arrays and high-throughput sequencing (HTS) follow a similar computational analysis workflow for the detection of CNAs: preprocessing of raw data, copy number profile segmentation and CNA calling, based on the average values of the resulting segments. The first step, preprocessing, is vital for improved CNA detection and is often underrated [10]. For HTS data, this step starts with read summarization, which involves counting the number of reads that fall within genomic windows, typically non-overlapping and fix-sized. The result is a window read count (RC) profile, which is a proxy to the true copy number profile. Some reads, such as those with low mapping quality [11], can be filtered during summarization, while whole windows can be filtered afterwards. Preprocessing may continue with normalization, which corrects for technical or biological factors that confound the true copy number profile, mainly the GC content [12]. Normalization against a matched normal sample allows a better correction of confounding factors [13] and reduces the need for filters, but it is not always available [14], hence the relevance of optimal filtering and GC content bias correction.

Here, we present a user-friendly and highly-parallelized R package, called *seqCNA*, which allows an integrated copy number analysis workflow. The package includes novel methodology on (i) window filtering, reducing false positives in comparison to assessed existing methods, and (ii) GC content correction, improving profile quality, especially under great read coverage and high correlation between GC content and copy number.

## Implementation

*seqCNA* is available as an R package through the Bioconductor project [15]. It depends on the GLAD [16], adehabitatLT [17], doSNOW [18] and *seqCNA.annot* R packages, which are automatically downloaded from the Bioconductor and CRAN [19] repositories as needed. The *seqCNA.annot* companion package contains annotation on GC content, mappability and presence of common CNVs for the included genome builds, enabling several optional steps of the analysis.

An integrated read summarization function, *seqsumm*, written in C++ and interfacing with the R code through Rcpp, makes *seqCNA* the only tool required to obtain copy number profiles from SAM alignment files (SAMtools [20] is necessary to read BAM files). The subsequent functions in the package return visual feedback throughout the analysis and require little parameterization. A vignette with a worked example and detailed help on functions and parameters are included within the package.

## Results and discussion

### Workflow

The *seqsumm* function summarizes read counts into windows of the selected size, but it also classifies paired-end mapping (PEM) reads based on their SAM flags - which consider read pairing, separation and orientation - and calculates mean window mapping quality, enabling two of the five window filters available in *seqCNA*. The first filter involves PEM read classification, which has previously been used to select reads prior to summarization [21] and to detect the limits of structural genomic rearrangements, including CNVs [22]. Improper reads are considered those that are not in read pairs with correct separation and orientation. We saw that genomic windows with an elevated proportion of improper reads tend to be outliers in the RC profile, probably indicating the presence of intra-window structural polymorphisms. If these windows are not of interest, they can be filtered by setting a maximum proportion of improper reads within each window. Second, directly filtering low mapping quality reads reduces the signal-to-noise ratio (SNR) of the RC profile [23] so, instead, *seqCNA* provides a filter that discards windows, based on the mean mapping quality of proper reads. A third filter, the trimming filter, removes windows with extreme RC values, with the distinctive feature that a prior correction against GC content is performed, avoiding trimming extreme RCs that are only due to extreme GC content. The remaining two filters discard windows with the presence of common CNVs described by Altshuler et al. [24] and low mappability, where the mappability of a window reflects the uniqueness of 35-nucleotide long sequences within it [25].

**Figure 1:**
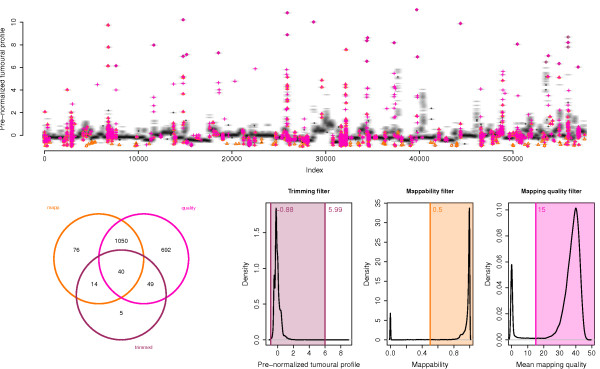
**Window filtering on HCC1143 sample’s RC profile.** Visual assessment of the filtering performed by seqCNA on sample HCC1143. The profile (top) shows where the filtered windows lie within it. Mappability-filtered windows are marked as orange triangles; mapping quality-filtered, as magenta crosses; and trimming-filtered, as purple dots. The Venn diagram shows the overlaps among the windows filtered by the three applied filters. The plots on the right depict the density maps on which thresholds delimit the windows to be discarded based on the different filters.

A matched normal sample is preferable (but not necessary) for the PEM-based, mapping quality and trimming filters, in order to prevent biases that arise due to the presence of CNAs. For instance, some extreme RCs on an unpaired tumoural profile can be due to CNAs and should be kept, so the process of trimming should be able to spot them to avoid their filtering. For the matter, the trimming filter in *seqCNA* uses an algorithm based on the Wald-Wolfowitz runs test to tell apart CNAs from outliers on unpaired tumoural profiles. It measures the randomness of the position of those windows with RC above a certain threshold, where the higher the threshold, the greater the randomness due to outliers. The threshold is set where a sudden change in randomness occurs due to the inclusion of adjacent windows, not likely to be outliers (see Additional file 1, Automatic trimming Section, for more details). The five filters are independent and are applicable based on availability of PEM reads, matched normal sample and genome build annotation. While each filter targets windows with a specific behavior, many windows are captured by more than one filter, increasing the filtering robustness (see Figure 1).

The next step of the analysis is normalization, which accounts for confounding factors in the RC profile. If a matched normal sample is not available, normalization involves GC content bias correction, which removes a great part of the observed bias. The described relationship between GC content and RC [12] is generally non-linear, so, until recently, GC content correction has been tackled through local regression (LOESS) or polynomial fitting [10, 11, 26]. Such approach is adequate for genomes where (i) gains and losses account for a small fraction of the genome or they are rather balanced and (ii) GC content distribution is similar among regions with different copy numbers, but this is not typically the case of cancer genomes, where a linear relationship between copy number and GC content produces a bias in the regression. The GC correction algorithm in FREEC [14] tries to improve mere regression by looking for the GC content curve of the main copy number, sampling window densities at specific GC content levels. Thus, FREEC is able to handle the possible correlation between copy number and GC content, as long as the correlation does not affect the convergence of the algorithm towards the main copy number. Furthermore, such algorithm works under the assumption of known sample ploidy and proportionality between RC and copy number, which may be shifted by the presence of subclones in the cell population.

**Figure 2:**
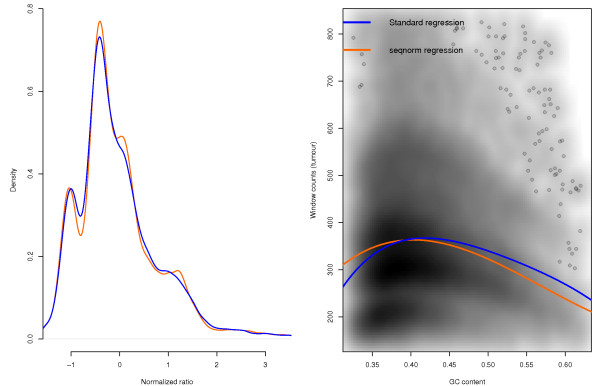
**GC correction of HCC1143 sample’s RC profile.** GC correction results over the HCC1143 sample’s RC profile. (Left) Density plot of the normalized RC profiles. The density of the seqnorm-corrected profile distinguishes better the different copy numbers in the mixture. (Right) Read counts depending on the window’s GC content, with greater density as darker grey. In blue, regression line estimated through the typical approach. In orange, regression line estimated by seqnorm.

The approach we propose for GC bias correction, called *seqnorm*, accounts for the correlation between copy number and GC content independently of sample characteristics. textitseqnorm is a two-iteration algorithm, with a first pass regression that removes much of the GC bias and a second step that accounts for the correlation between GC content and copy number before a second pass regression. While the first regression is sensitive to the correlation and can, therefore, under- or over-correct the GC curve, the GC bias generally decreases. Afterwards, GLAD [16] produces a segmented profile in a way that segments represent the maximal neighbourhoods in which the local constant assumption of the statistical model holds. Such property is interesting because low intra-segment variability is key to the second regression, which is applied segment-wise. Namely, segments with the highest RC variability, as well as those spanning few windows, may not provide robust enough fits. In turn, those with little GC content variability do not allow estimating the effect of extreme GC content. Therefore, segments undergo a selection process (see Additional file 1, *seqnorm* Section, for more details). Centering the selected segments removes the read count differences due to copy number changes, essentially removing the undesired correlation. Thus, the subsequent segment-wise regressions provide good approximations to the genome-wide effect of GC content on read counts without the bias that emerges from the correlation and their median gives a robust estimate of the true effect (see Figure 2). Although devised to improve GC content normalization, *seqnorm* can also be used to normalize against matched paired normal.

**Figure 3:**
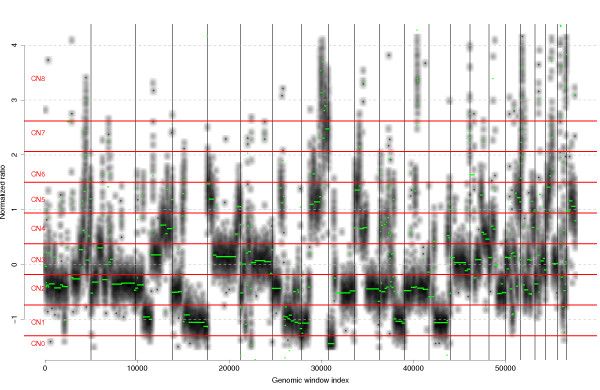
**Copy number calling of HCC1143 sample’s segmented RC profile.** Visualization of the GC corrected and segmented profiles of sample HCC1143, together with the thresholds that delimit copy number calls. Chromosomes are delimited by black vertical lines. The GC corrected profile is shown as a black-coded density map, the segmented profile is shown in green and the thresholds are marked in red.

Existing tools for CNA detection on HTS data perform a basic preprocessing and put their focus on the copy number calling step, taking advantage of allele-specific information [14, 27, 28] and automatically predicting copy number profiles [10, 14, 27]. On the other hand, *seqCNA* focuses on preprocessing and provides additional simple methods to obtain the final copy number profiles. Hence, the analysis is completed by segmenting the *seqnorm*-corrected profile with GLAD and, through visual assessment, defining copy number limits (Figure 3).

### Methodology assessment

For the methodology assessment, we used data from human samples according to the Declaration of Helsinki, the European Guidelines on Good Clinical Practice, relevant national and regional authority requirements and Hospital Clinico San Carlos’s Clinical Investigation Ethics Committee (Madrid, Spain). Informed consent was obtained from every subject.

In a recent comparison [29], we found GAP [30] to be the best performing CNA-detecting method on SNP-array data. Knowing that, we compared the results from *seqCNA*, GAP and state-of-the-art CNA-detecting methods on HTS data, namely FREEC [14], CNAnorm [10] and Patchwork [28], over two colon cancer samples we hybridized on SNP-arrays and paired-end sequenced (raw data is deposited at the European Genome-phenome Archive (EGA) under accession number EGAS00001000558). *seqCNA*’s copy number profiles were the closest ones to GAP’s (Additional file 1: Table S3) and presented the lowest departure from consensus profiles: 0.04% in both cases (Additional file 1: Table S2). The novel PEM-based and mapping quality filters are the main reason behind the reduced false positive rate in comparison to FREEC and CNAnorm (Additional file 1: Figures S1 and S3).

In order to assess *seqnorm*’s performance, we built a semi-simulated dataset from the combination of the copy number profiles of 676 cell-lines [31], determined using PICNIC [32], and the RC variability of 7 non-tumoural samples (see Additional file 1, Simulated dataset Section, for more details). On the semi-simulated dataset, real cell-line [33] and prostate cancer samples [34] the median signal-to-noise ratio (SNR) improvement with respect to typical regression ranged from 1% to 4% (Additional file 1: Figure S9), with a maximum improvement between 27% and 77%, where 2% already yields a visibly more defined density map (see Figure 2). We investigated the factors that affect this improvement and saw that it directly depends on: (i) the SNR of the RC profile and (ii) the correlation between GC content and copy number, which tends to be greater when the top main copy number (spanning at least 5% of the genome) is 4 or 5 and there are between 3 and 4 distinct main copy numbers (Additional file 1: Figure S11).

### Future development

As of the initial release, the annotation package covers the human genome with builds *hg18* and *hg19*. In the future, we aim at extending the package to include further genomes and builds based on users’ necessities.

We are also pondering possible extensions to the main package, including relevant region annotation and support for side-by-side displaying of copy number profiles in existing databases. Increased automation is also plausible, especially in the final calling step, but we reckon that improved methodology needs to be developed in this regard in order to replace human judgment. In general, while GC bias correction is a mature issue, we expect the filtering and calling steps to see further developments. Specifically, additional intelligent filter threshold selection and multifactorial filtering are issues that remain open.

## Conclusion

We have presented *seqCNA*, a tool that allows integral analyses for the detection of CNAs in HTS tumoural data and provides relevant advancements in the preprocessing steps. Namely, it incorporates a novel normalization method, *seqnorm*, which significantly improves the performance of typical regression, especially on samples with high SNR (e.g. due to greater coverage) and under high correlation between GC content and copy number. The tool also incorporates novelties for the filtering of windows in RC profiles - thus reducing the amount of false positives, including a PEM-based filter, a method that automatically sets trimming thresholds and a sensible window filter that replaces the removal of low quality reads.

## Availability and requirements

**Project name:** seqCNA

**Homepage:**http://www.bioconductor.org/packages/devel/bioc/html/seqCNA.html

**Operating system(s):** Platform independent

**Programming language:** R

**Other requirements:** SAMtools (only if using BAM files)

**License:** GPL-3

**Any restrictions to use by non-academics:** None

## Electronic supplementary material

Additional file 1: **Supplementary methods, figures and tables.** (PDF 2 MB)
