# Supplementary material for: seqCNA: an R package for DNA copy number analysis in cancer using high-throughput sequencing
Source: BMC Genomics. 2014 Mar 5;15(1):178. doi: 10.1186/1471-2164-15-178 (PMC4022175; doi:10.1186/1471-2164-15-178)
Supplement: Supplementary file 1 — Additional file 1: Supplementary methods, figures and tables. (PDF 2 MB) [file 12864_2013_7021_MOESM1_ESM.pdf]

Additional file 1

seqCNA: an R package for DNA copy number analysis in cancer using high-throughput sequencing

David Mosén-Ansorena [1]*, Naiara Tellería [2], Silvia Veganzones [3], Virginia de la Orden [3], María Luisa Maestro [3] and Ana M. Aransay [1]

[1]CIC bioGUNE & CIBERehd, Technologic Park of Bizkaia, Building 502, 48160 Derio, Spain
[2]Dominion Pharmakine S.L., Technologic Park of Bizkaia, Building 801, 48160 Derio, Spain
[3]Clinical Analyses Service at the San Carlos Clinical Hospital, Martin Lagos, 28040 Madrid, Spain

1. SAMPLES

In this section, we describe the cell-line and tumoural samples used to assess the filtering and normalization methods in *seqCNA*. Specifically, we used two in-house colon cancer samples to assess the filtering. Then, to assess normalization, we used the HCC1143 cell-line sample described by Chiang et al. (Chiang *et al.*, 2008) and the prostate cancer sample with code PR-3027 from the study by Berger et al. (Berger *et al.*, 2011).

Finally, certain aspects of additional samples were used to generate synthetic data on which to also assess normalization. These samples are:

- Four samples from cell-lines: COLO-829BL and COLO-829 (Pleasance *et al.*, 2010), NCI-H2171 (Campbell *et al.*, 2008) and HCC1143 (Chiang *et al.*, 2008). Their GC curves summarized at 50Kb, as obtained by Boeva et al. (Boeva *et al.*, 2011), were used.
- Seven non-tumoural samples: PR-0508, PR-0581, PR-1701 and PR-1783 from Berger et al. (Berger *et al.*, 2011), HCC1954BL from Chiang et al. (Chiang *et al.*, 2008), and samples 4077 and 10002 introduced in this study. The variability of their profiles summarized at 50Kb was used.
- The copy number profiles from 746 cell-line samples, as determined by Bignell et al. (Bignell *et al.*, 2010) from Affymetrix SNP6.0 array data.

See Additional file 1: Table S1 for details on all sequenced samples used in this study.

1.1 IN-HOUSE COLON CANCER SAMPLES

We hybridized and sequenced two samples coming from colon cancer biopsies, with codes 4076 and 10001. This enabled us to compare analysis methods for SNP-arrays and high-throughput sequencing. Paired-end mapping reads enabled us to apply and assess the performance of the PEM-based filter. We also hybridized and sequenced the corresponding matched normal samples, with codes 4077 and 10002.

The human samples were obtained and used according to the Declaration of Helsinki, the European Guidelines on Good Clinical Practice, relevant national and regional authority requirements and Hospital Clinico San Carlos's Clinical Investigation Ethics Committee (Madrid, Spain). Informed consent was obtained from every subject.

a) Whole Genome SNP Genotyping

After checking their integrity and concentration, the four aforementioned DNA samples were processed following the *Infinium HD Assay* manual of Illumina Inc., and amplified DNAs were

hybridized on *Human 660W-Quad BeadChips*. Intensities were obtained in a Bead Array Station (Illumina Inc.) and decoded with GenomeStudio software.

b) High Throughput Sequencing

Sequencing Libraries were prepared according to *TruSeq DNA sample Prep v2 Low throughput* protocol of Illumina Inc. and quantified by qPCR. Each library was paired-end sequenced in 1 lane of a HiScanSQ flow cell (Illumina Inc.) at 16pM for 50 cycles. The reagents used for these sequences were TruSeq PE Cluster Kit v3 - cBot – HS, HiSeq Flow Cell v3 and TruSeq SBS Kit v3. We used HCS_1.5.15 and the RTA version 1.13.48 for data collection.

Additional file 1: Table S1. List of all high-throughput sequenced samples together with basic information and their use in this study.

Total reads (M) is the amount of reads obtained per sample ranged, in millions, from few tens for some cell-line samples to around one thousand. Specifically, samples with three levels of read depth were used: 26-45 million reads, 84-106 million reads and 572-1,069 million reads. Read length (bp) ranged from 29 to 101 base pairs. The sequencing type and alignment strategy were single-read for some samples and paired-end for others. Tumour purity, which indicates the proportion of estimated actual cancer cells in each sample, was between 0.47 and 0.74 for tumoural samples and 1 for cell-lines, with only cancer cells. On the reads aligned by us, we found no significant difference between BWA (Li and Durbin, 2009) and Bowtie (Langmead *et al.*, 2009) alignments. Presented results are based on BWA alignments with default parameterization or MAQ (Li *et al.*, 2008), for the samples from Berger *et al.*, 2011. Samples were used to assess the filters (Filters) and *seqnorm* normalization (Norm) in *seqCNA*, or to build synthetic data (Synth) in order to further test *seqnorm* normalization.

Sample	Original study	Description	Total reads (M)	Read length (bp)	Strategy	Tumour purity	Ref. genome	Aligner	Use
4076		Colon cancer	106	50	Paired-end	0.6	Hg19	BWA	Filters
10001		Colon cancer	92	50	Paired-end	0.47	Hg19	BWA	Filters
4077		Colon normal tissue	97	50	Paired-end	NA	Hg19	BWA	Synth
10002		Colon normal tissue	84	50	Paired-end	NA	Hg19	BWA	Synth
HCC1143	Chiang *et al.*, 2008	Breast adenocarc. cell line	45	36	Single read	1	Hg19	BWA	Norm, Synth
COLO829BL	Pleasance *et al.*, 2010	Lymphoblastoid cell line	26	50	Single read	NA	Hg19	BWA	Synth
COLO829	Pleasance *et al.*, 2010	Melanoma cell line	27	50	Single read	1	Hg19	BWA	Synth
NCI-H2171	Campbell *et al.*, 2008	Small-cell lung cancer cell line	36	29, 36	Single read	1	Hg19	BWA	Synth
HCC1954BL	Chiang *et al.*, 2008	Lymphoblastoid cell line	31	36	Single read	NA	Hg19	BWA	Synth
PR-3027	Berger *et al.*, 2011	Primary prostate tumour	927	101	Paired-end	0.74	Hg18	MAQ	Norm
PR-0508	Berger *et al.*, 2011	Normal tissue	957	101	Paired-end	NA	Hg18	MAQ	Synth
PR-0581	Berger *et al.*, 2011	Normal tissue	572	101	Paired-end	NA	Hg18	MAQ	Synth
PR-1701	Berger *et al.*, 2011	Normal tissue	1,069	101	Paired-end	NA	Hg18	MAQ	Synth
PR-1783	Berger *et al.*, 2011	Normal tissue	914	101	Paired-end	NA	Hg18	MAQ	Synth

2. ANALYSIS OF IN-HOUSE COLON CANCER SAMPLES

Sequencing reads were aligned with the original BWA (Li and Durbin, 2009) with default parameters. Afterwards, the BAM files were summarized into 50Kbps windows using the summarization tool integrated in *seqCNA*, obtaining 106M and 92M mapped reads for the 4076 and 10001 samples, respectively.

In *seqCNA*, filter thresholds were set at 0.85 for mappability, 30 for mapping quality, 1% for improper read ratio, 0.05% for extreme values in the lower quantile and automatic selection for extreme values in the upper quantile. There was a considerable overlap between the mappability and mapping quality filters, as expected, and between the PEM-based filter and the others, supporting the usefulness of such novel filter (Additional file 1: Figures S1 and S3).

Copy number calling was made through visual assessment. For the normalized ratios of tumoural sample 4076, thresholds for copy numbers were established at intervals of 3.0 starting at -4.6, so that windows between -4.6 and -1.6 were assigned copy number 0; windows up to 1.4, copy number 1; and so on. For sample 10001, thresholds for copy numbers were established at intervals of 1.95 starting at -3.1, so that windows between -3.1 and -1.15 were assigned copy number 0; windows up to 0.8, copy number 1; and so on.

Essentially, sample 4076 presented a duplication of chromosome 12 and a heterozygous loss of the q arm in chromosome 13 (Additional file 1: Figure S2). The copy number profile of sample 10001 was a bit more complex (Additional file 1: Figure S4), with whole duplications of chromosomes 7 and 16, and heterozygous losses of chromosomes 14 and 18. Chromosomes 17 and 20 presented both gains and losses that spanned long chromosomal regions. Finally, there seemed to be population subclones in the sample, a few of which presented a duplication of chromosome 13.

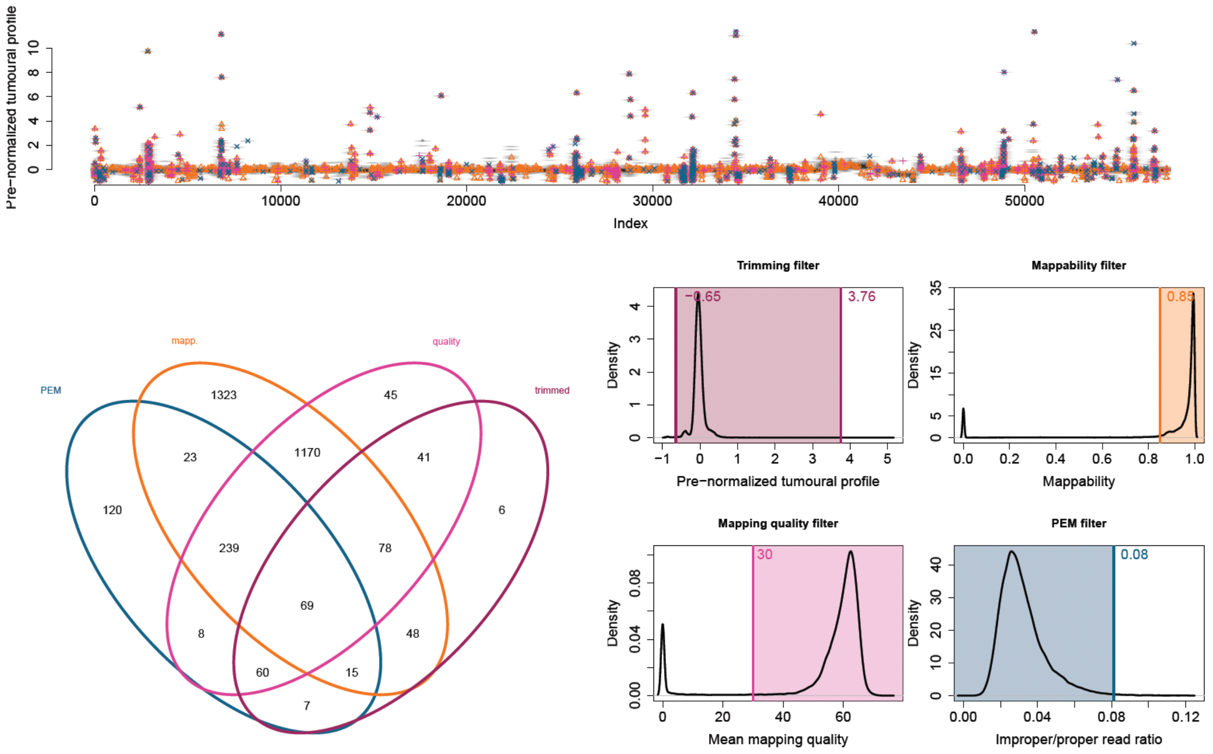

Additional file 1: Figure S1. Visual assessment of the filtering performed by *seqCNA* on sample 4076. The profile shows where the filtered windows lie within it. Code: PEM-filtered windows, blue crosses; mappability, orange triangles; mapping quality, magenta plus signs; trimming, purple dots. (Left) Venn diagram with the overlaps among the windows filtered by the four applied filters. (Right) Density maps on which thresholds delimit the windows to be discarded based on the different filters.

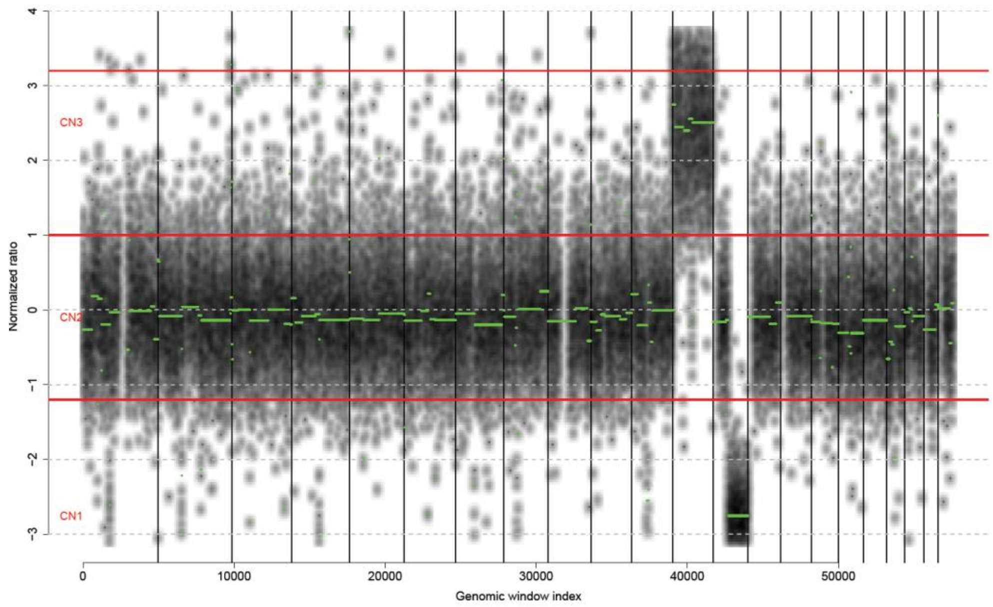

Additional file 1: Figure S2. Visualization of the normalized and segmented profiles of sample 4076, together with the thresholds that delimit copy number calls. Chromosomes are delimited by black vertical lines. The normalized profile is shown as a black-coded density map, the segmented profile is shown in green and the thresholds are marked in red.

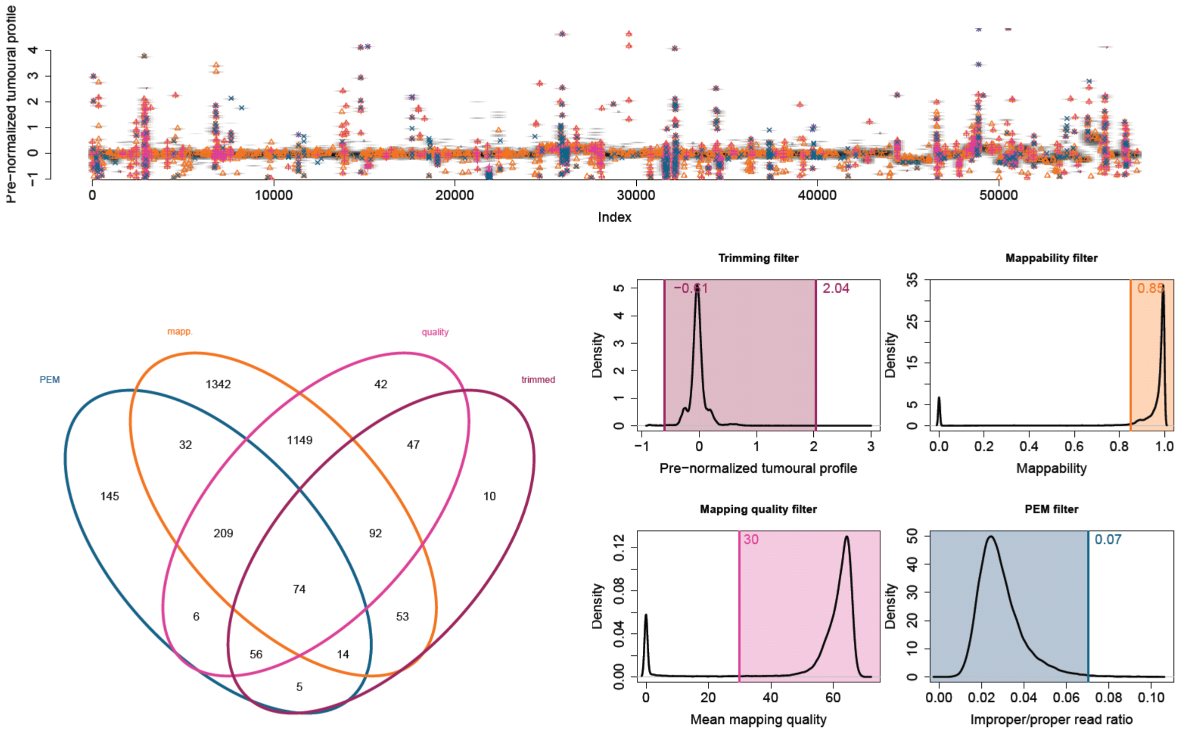

Additional file 1: Figure S3. Visual assessment of the filtering performed by *seqCNA* on sample 10001. The profile shows where the filtered windows lie within it. Code: PEM-filtered windows, blue crosses; mappability, orange triangles; mapping quality, magenta plus signs; trimming, purple dots. (Left) Venn diagram with the overlaps among the windows filtered by the four applied filters. (Right) Density maps on which thresholds delimit the windows to be discarded based on the different filters.

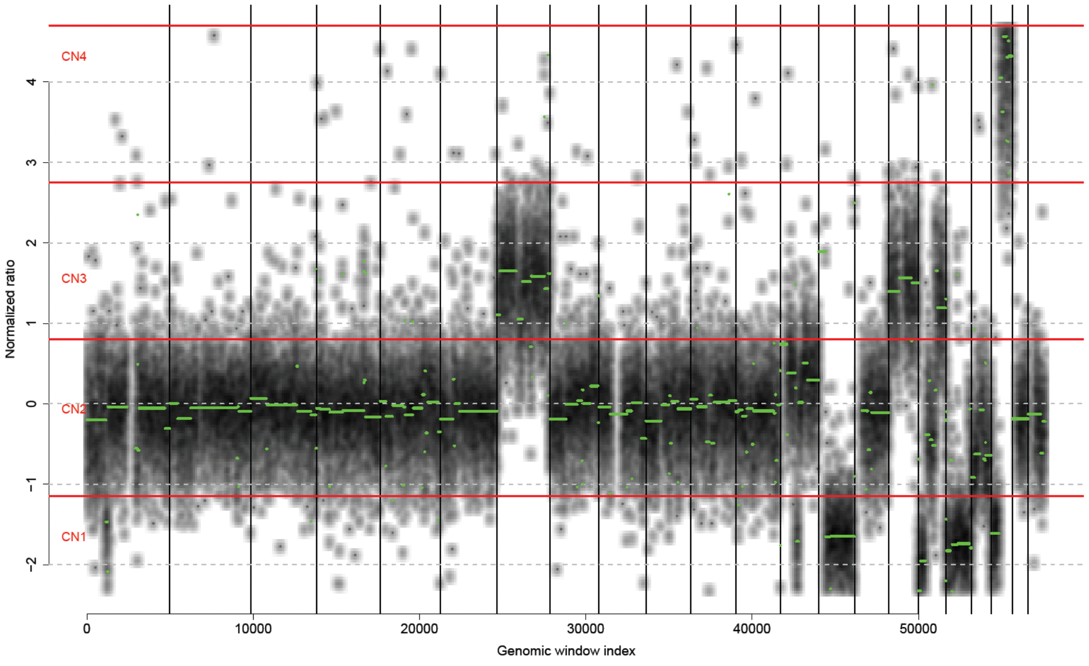

Additional file 1: Figure S4. Visualization of the normalized and segmented profiles of sample 10001, together with the thresholds that delimit copy number calls. Chromosomes are delimited by black vertical lines. The normalized profile is shown as a black-coded density map, the segmented profile is shown in green and the thresholds are marked in red.

3. FILTERING

The five filters available in *seqCNA* are based on PEM mapping, mapping quality, mappability, common CNVs and extreme read count (RC) values.

3.1 COMPARISON WITH OTHER METHODS

The two in-house colon cancer samples present little correlation between GC content and copy number. Additionally, their copy number profiles are simple, present little intra-tumour heterogeneity and are mainly diploid. Hence, GC normalization and copy number calling are simple enough so that filtering is the main reason for variation in results among CNA analysis methods for sequencing data. Namely, we assessed the following methods: GAP for SNP-array data (Popova *et al.*, 2009), and CNAnorm (Gusnanto *et al.*, 2012), FREEC (Boeva *et al.*, 2011) and *seqCNA*. Apart from *seqCNA*, only FREEC has the option to discard windows (by mappability).

The SNP-array data was adjusted with the GC correction tool in PennCNV (Wang *et al.*, 2007) and TumourBoost (Bengtsson *et al.*, 2010). Then, the LRR signal was normalized with the matched normal LRR. GAP was run with a segmentation step that disregarded the LRR, to avoid excessive segmentation. The window-wise profile was then built in such a way that windows take the most frequent value of the probes within them. Some windows did not contain any probes and, thus, were not assigned any value.

The alignment and summarization procedures on the sequencing data, together with *seqCNA* parameterization are described in the previous section. CNAnorm was run without any filtering and FREEC filtered windows with mappability lower than the default threshold 0.85. Finally, Patchwork (Mayrhofer *et al.*, 2013) was run with default parameters up until the final calling step, where the cn2 and delta parameters (indicating the CN2 position and the distance between CNs) were set to: (i) 0.87 and 0.36 for sample 4076, and (ii) 0.95 and 0.2 for sample 10001. These parameters resulted in the profiles with highest similarity to those of the other methods, and variations of up to 0.03 did not alter the yielded profiles.

In this study, we only considered autosomal chromosomes, for which a consensus copy number profile was built, where at least 4 out of the 5 methods had to make the same call in a window for it to get that value. Consensus profiles had 50,412 and 48,958 windows for samples 4076 and 10001, respectively. See Additional file 1: table S2 for dissimilarities between profiles.

Additional file 1: Table S2. Copy number calls against consensus profile for samples 4076 and 10001.

SAMPLE 4076	Same call	Different call		SAMPLE 10001	Same call	Different call
seqCNA	50,331	21 (0.04%)		*seqCNA*	48,886	19 (0.04%)
CNAnorm	50,201	211 (0.42%)		CNAnorm	48,101	857 (1.75%)
FREEC	50,367	45 (0.09%)		FREEC	41,886	7,072 (14.45%)
Patchwork	49,670	551 (1.10%)		Patchwork	48,718	29 (0.06%)
GAP	49,356	290 (0.58%)		GAP	48,053	320 (0.66%)

Additional file 1: Table S3. Dissimilarity (in percentages) of copy number calls between methods for samples 4076 and 10001.

SAMPLE 4076	*seqCNA*	CNAnorm	FREEC	Patchwork	GAP
seqCNA	-	0.8	0.4	1.4	1.0
CNAnorm	0.4	-	1.2	3.6	3.1
FREEC	0.4	1.2	-	1.6	1.3
Patchwork	1.4	3.6	1.6		2.8
GAP	1.0	3.1	1.3	2.8	-

SAMPLE 10001	*seqCNA*	CNAnorm	FREEC	Patchwork	GAP
seqCNA	-	3.7	17.0	1.9	2.2
CNAnorm	3.7	-	19.3	4.4	6.6
FREEC	17.0	19.3	-	17.4	17.0
Patchwork	1.9	4.4	17.4		4.2
GAP	2.2	6.6	17.0	4.2	-

Pairwise comparisons show that *seqCNA*'s profiles present the lower dissimilarities to the other profiles, suggesting that they are closer to the true copy number profiles, while the rest diverge from them in different sets of windows (see Additional file 1: Table S3).

As a side note, the consensus and pair-wise comparisons show that FREEC has a problem with the calling of sample 10001.

Since samples 4076 and 10001 presented little correlation between GC content and copy number, normalization did not contribute to the improvement offered by *seqCNA*. Given that calling is trivial in *seqCNA*, it seems that the extra filtering is the factor that reduced the differences with respect to the other profiles, and has therefore the potential to reduce the false positive rate.

3.2 INDIVIDUAL FILTER ASSESSMENT

Given that some of the filters are novel, we wanted to estimate their effect on discarding windows that do not seem to require filtering. For the trimming filter, only the top quantile was considered, as very little trimming is usually necessary on the bottom quantile.

For the matter, FREEC and *seqCNA* were re-run without filtering on the data from the two tumoural samples 4076 and 10001 summarized at 50Kbps, and another consensus profile was built where at least 3 out of 4 methods (FREEC, seqCNA, GAP and CNAnorm) had to make the same call in a window for it to get that value. We only considered windows with GC content information and with a consensus value. If a window with consensus value was discarded by a filter, it was considered to be a filter's false positive. Hence, in this assessment, the false positive rate (FPR) of a filter is defined as the proportion of windows it discards but whose values are called equally by the majority of the methods. The described FPRs, although not true estimates of the actual filters' FPRs, allowed the comparison of the performance among filters and degree of filtering.

As expected, greater filtering levels yielded higher FPRs, with the mappability and trimming filters being the ones with lower FPRs (Additional file 1: Figure S5). Nevertheless, for sample 4076, we observed a rapid increase in FPR for the trimming filter as more windows were filtered. This occurs because few outlier values are above those of windows with a copy number gain, and these latter quickly begin to be discarded as the threshold lowers.

The PEM-based and mapping quality filters offer sensible FPR curves up to around 1% filtering, with around 20% FPR at that level. Still, if controlling FPR is imperative, increasing their filtering can be helpful, as they target windows otherwise overlooked by the mappability filter. For instance, regions in windows 15190 to 15210, 21815 to 21925 and 50860 to 50915 appear as apparent losses in all samples we have studied, and cannot be discarded with the mappability filter (data not shown). Notably, the second mentioned region corresponds to the major histocompatibility complex (MHC) region in chromosome 6.

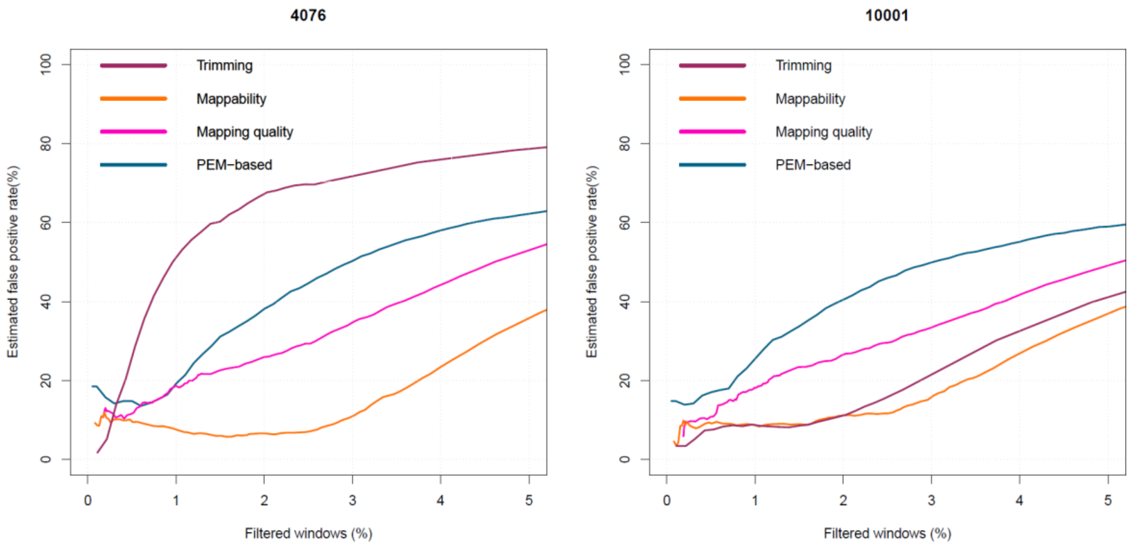

Additional file 1: Figure S5. Comparison of estimated falsely filtered windows by filter type and depending on the percentage of filtering, for samples 4076 and 10001.

3.3 AUTOMATIC TRIMMING

Some genomic windows may present clearly outlier RC values which are too extreme to be considered due to true copy number changes. The trimming filter discards them based on a threshold.

To minimize the filtering of actual copy number polymorphisms, it is best to filter based on the RC profile of a matched normal sample. However, setting the threshold here can be quite arbitrary, given that RCs follow a unimodal distribution. Thus, it is a matter of weighing the requirements on false positives and false negatives. If the trimming needs to be performed on a tumoural profile, the approach should be different for the lower and upper sides of the distribution.

On one hand, copy number gains can be mistaken for outliers with high values but, in this case, adjacent windows with high and similar RCs tend to be true gains, so we can use this information to tell them apart from isolated outliers, as long as gains span several adjacent windows. This adjacency pattern of true gains can be used to determine an optimal top threshold selection. Let us define a run as an adjacent set of windows which are all either selected or not selected for filtering given a certain

threshold. As we lower the threshold, we expect to include in the filtering more and more true gains, which decrease, proportionally to their size, the number of runs. Hence, the number of runs sees a significant decrease, as evaluated through the Wald-Wolfowitz runs test, when clear gains begin to be selected. We wish to notice, however, that there can be adjacent windows with high RCs that are not true gains. This phenomenon typically appears on the centromere areas as is not intended to be captured by the trimming filter. Instead, the PEM-based and mapping quality filters are appropriate for this task.

On the other hand, extremely low RC in some regions is due to low mapping quality, so virtually no windows would be filtered with the aforementioned approach on the lower side of the distribution. The threshold with the aforementioned approach would be set practically. Nevertheless, most of these outliers can be identified through the filters for mappability, mapping quality and PEM ratio.

We implemented a function in *seqCNA* that calls the Wald-Wolfowitz runs test (Calenge, 2006) at a range of RC quantile thresholds up to 2%. After the curve of test scores is smoothed, the point of maximum curvature is selected as the optimal threshold. This point lies in the RC value before gains are selected for filtering (see Additional file 1: Figure S6 for an example).

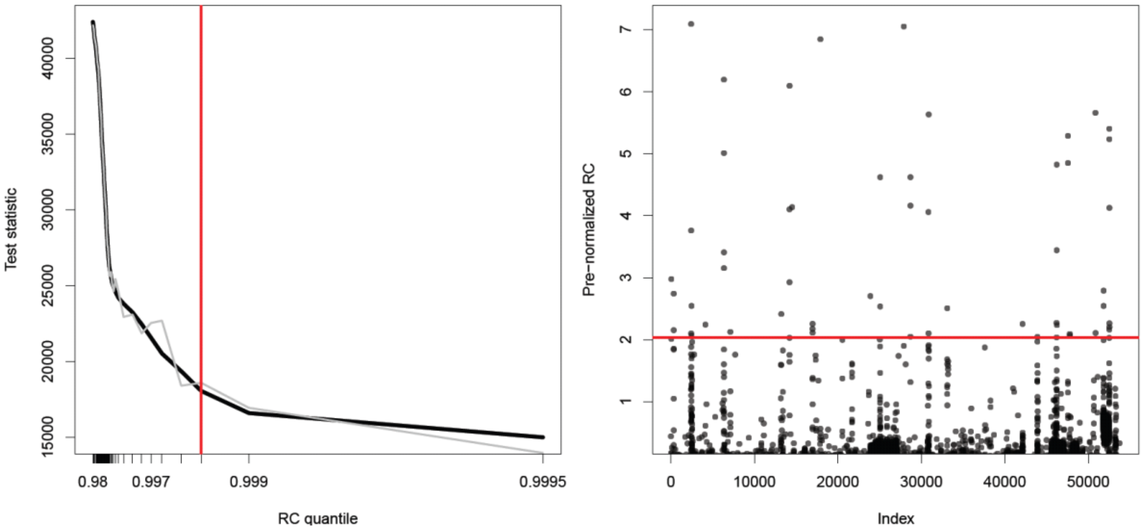

Additional file 1: Figure S6. Automatic trimming threshold selection based on the Wald-Wolfowitz test statistics for the 10001 sample. Left: value of the test statistic depending on which top pre-normalized RC quantile is selected (grey), with smoothed progression of the values (black) and quantile at which the smoothed progression presents the maximum curvature (red line). Right: the trimming threshold is placed at the aforementioned quantile.

4. NORMALIZATION WITH *SEQNORM*

The correlation between GC content and copy number arises because of an uneven distribution of GC content among regions with different copy numbers. The GC content normalization approach proposed in *seqnorm* is a two-iteration algorithm, with a first pass regression that removes much of the GC bias and a second step that accounts for the correlation between GC content and copy number before a second pass regression. In the current implementation, the regression method can be chosen among locally weighted (LOESS) and polynomial, which in turn can be cubic or quadratic. As pointed out by Boeva et al. (Boeva *et al.*, 2011), adding further complexity to the polynomial regression is not necessary.

Before normalization, if a RC profile is dense and contains many windows, *seqnorm* automatically summarizes windows into longer windows. This has the benefit of not only reducing computation time, but also increasing the signal-to-noise ratio. In turn, better signal-to-noise ratio implies easier segmentation and longer segments, which provide greater robustness in the normalization process.

While standard regression is sensitive to the correlation between GC content and copy number, and can, therefore, under- or over-correct the GC curve, the bias after this regression is generally lower. Hence, after the possible reduction in the number of windows, the RC profile undergoes an initial correction with standard regression, likely to remove much of the GC bias.

Afterwards, the adaptive weights smoothing in GLAD produces a segmented profile in a way that segments represent the maximal neighbourhoods in which the local constant assumption of the statistical model holds. Such segment property is interesting because low intra-segment variability is key to subsequent segment-based regression. In the current implementation, GLAD is run with default parameters but allows changing the lambda parameter, which is the penalty term used during the optimization of the number of breakpoints step. The primary (post-normalization) segmentation uses the same method and parameterization options.

Segments with high read count variability may be the result of under-segmentation and contain regions with slightly different copy numbers, possibly due to the presence of subclones in the sample population. Such segments are not suitable for the removal of the correlation between GC content and copy number. In turn, segments with few windows do not provide enough robustness and those with little GC content variability do not allow estimating the effect of extreme GC content. Therefore, segment selection keeps only those ones that are most likely to provide good regression estimates. An initial selection is made with segments that lie at the intersection of those with most disperse GC content, less disperse RC and greater length. Too few segments do not provide enough robustness to the subsequent regression, as the medians are calculated from few values. On the other hand, too many segments may add extra noise in the regression and the fitting task becomes computationally expensive. Thus, thresholds are relaxed or restricted until the number of segments lies between 7 and 35 (chosen empirically). A simple copy number profile or a low intersection among the three restrictions may result in a set of less than 7 segments. In this case, *seqnorm* falls back to standard regression and does not perform the second, segment-based, regression. Nevertheless, usually, there is a strong intersection among the restrictions, as longer segments tend to converge towards an average normalized RC range and greater GC domain (Additional file 1: Figure S7).

In order to remove correlation between GC content and read count differences due to copy number changes, the selected segments are centered so that they have the same value at the median GC content. Then, the initially chosen regression is applied to each segment. The segment-wise regressions provide good approximations to the genome-wide effect of GC content on read counts

without the bias that emerges from the correlation and their median gives a robust estimate of the true effect (see Figure 2 in the main text).

The robustness of the final estimate depends on the local window density along the GC content distribution. Therefore, the estimation for regions with extreme GC content, which are less common, may not be as accurate, but the estimation for most of the genome windows, with near-average GC content, is very robust (Additional file 1: Figure S8).

Although devised to improve GC content normalization, *seqnorm* can also be used to normalize against matched paired normal. In this case, the regression intercepts are automatically set at zero.

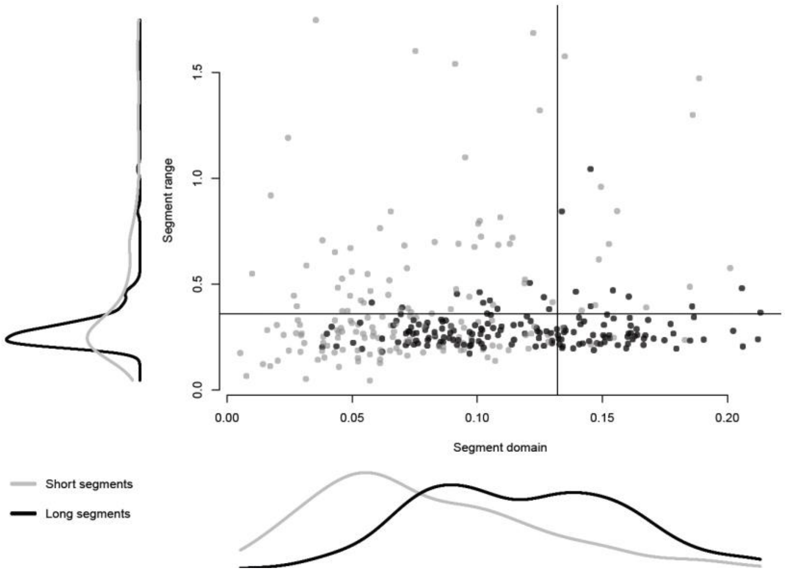

Additional file 1: Figure S7. Segment domain versus segment range for cell-line HCC1143 in 50Kb windows. Grey points represent segments with a length below the median and black points, segments with length above it. The left and bottom plots depict the density of these two groups in terms of range and domain, respectively. The horizontal and vertical lines in the main plot divide the range and the domain into their 0.75-0.25 quantiles. Selected segments lie in the lower-right quadrant. Notice how range variability is smaller for longer segments. Also, as expected, longer segments span a greater domain.

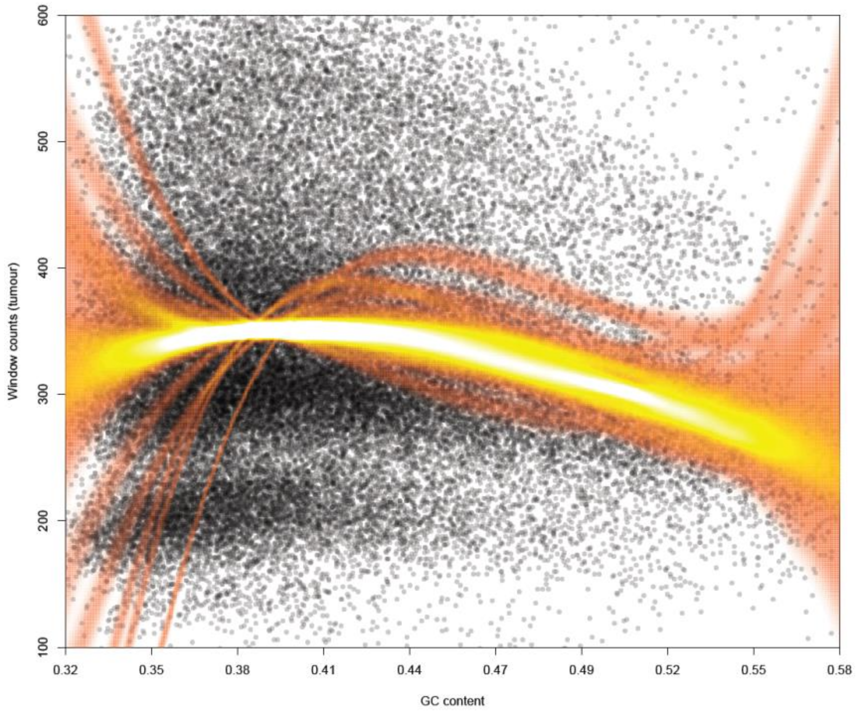

Additional file 1: Figure S8. GC content versus read count in 50Kb windows for HCC1143. The density of filtered segment fits is overlaid. Whiter and more opaque colour represents greater density (in contrast to more orange and translucent) and, therefore, better median fit estimation. GC content intervals with greater density tend to offer more reliable estimates.

4.1 SIMULATED DATASET

In order to compare *seqnorm* with standard regression, we generated a synthetic dataset that simulates summarizations of high-throughput sequencing reads at 50Kb, a resolution that is appropriate for normalization of a wide range of read count levels (see Results on real samples). The synthetic data arises from the conjunction of a set of models: copy number, normal cell contamination, noise and GC content. For the copy number model, we used the segmentation data determined using PICNIC (Greenman *et al.*, 2010) for 746 cancer cell lines (Bignell *et al.*, 2010). We filtered out 70 of them for presenting a near diploid genome (no copy numbers other than copy number 2 represented more than 5% of the genome) and kept only the autosomal chromosomes. Normal cell contamination was modeled with a simple formula:

$m = (n-2)*(1-c)+2$

Where n is the copy number, c is the degree of normal cell contamination (between 0 and 1) and m is the copy number of the mixture of tumoural and normal cells. GC curves were generated by tracing of a fourth degree polynomial through key knots, which were selected so that each curve resembled the GC curve observed for four real samples (COLO-829BL, COLO-829, NCI-H2171, HCC1143) (Boeva *et al.*, 2011). The fact that the curves and the regressions performed by the compared methods arise from the same model avoids possible differences in the results due to the regression step. Finally, noise is empirically modeled. We selected several normal samples that range from low to very high coverage and checked for long spanning copy number polymorphisms. Seven of these

samples were further selected: HCC1954BL (Chiang *et al.*, 2008), the matched normals of 4076 and 10001 (4077 and 10002), and the matched normals of PR-0508, PR-0581, PR-1701 and PR-1783 (Berger *et al.*, 2011). After their profiles were scaled and centered, the amount of noise observed in a sample would be inversely proportional to window size and depth of coverage. Therefore, given that we fixed window size at 50Kb, we applied greater noise to simulate lower depth of coverage.

For this problem, signal-to-noise ratio (SNR) is a measure of quality that indicates how discernible the copy number lines are from the rest. Specifically, the unit of signal is defined as the distance between two consecutive copy numbers under null normal cell contamination. The unit of noise is defined in terms of the standard deviation of the whole genomic signal. Lower read count increases the noise, while normal cell contamination makes the genomic signal converge towards the read count expected for diploid regions, hence decreasing the signal. In contrast to these two factors, intra-tumour heterogeneity does not affect signal or noise genome-wide, but region-wise. The read count of regions with intra-tumour heterogeneity is the weighted average of the copy number mixture, thus increasing noise from a genome-wide perspective. Regardless, the source of SNR reduction is of little importance for the performance of normalization methods. Still, our simulated dataset combines both normal cell contamination and raw noise (as a proxy to decrease in read count) as the sources of noise. In turn, because it is hard to decipher to what extent intra-tumour heterogeneity affects real samples and what the actual subclone composition is (Parisi *et al.*, 2011), we opted not to model it. We selected a variety of combinations of normal cell contamination and noise levels to produce SNR ranges that we classified as low (SNR 3.6-6), medium-low (6-8.4), medium-high (8.4-12.8) and high (12.8-18). The lowest SNR range reflects the level where normal cell contamination is above 50% and read count is rather low. On the other end, beyond the SNRs considered, signal quality is so high that improvement in normalization is irrelevant and one should not expect better CNA profiling thanks to it.

a) RESULTS ON THE SIMULATED DATASET

The improvement provided by *seqnorm* with respect to typical regression depends on two factors: (1) the SNR of the raw RC profile, (2a) the correlation between GC content and copy number, which in turn depends on the number and distribution of main distinct copy numbers in the profile.

The SNR of the raw RC profile steadily increases *seqnorm*'s improvement (Additional file 1: Figure S9). Consequently, *seqnorm*'s advantage becomes greater as higher coverages are considered. The correlation between GC content and copy number is measured by the F statistic. As the F-value increases, the improvement tends to be greater (see Additional file 1: Figure S10 for an example), but variability in the expected improvement also increases (Additional file 1: Figure S9). Finally, another way to see the correlation is to look at how the copy numbers in the profile are organized. Too few distinct main copy numbers (up to two) imply little opportunity to see correlation, but too many (from five on) make the linear relationship dilute, as their GC contents have lower chance to shift linearly (Additional file 1: Figure S11).

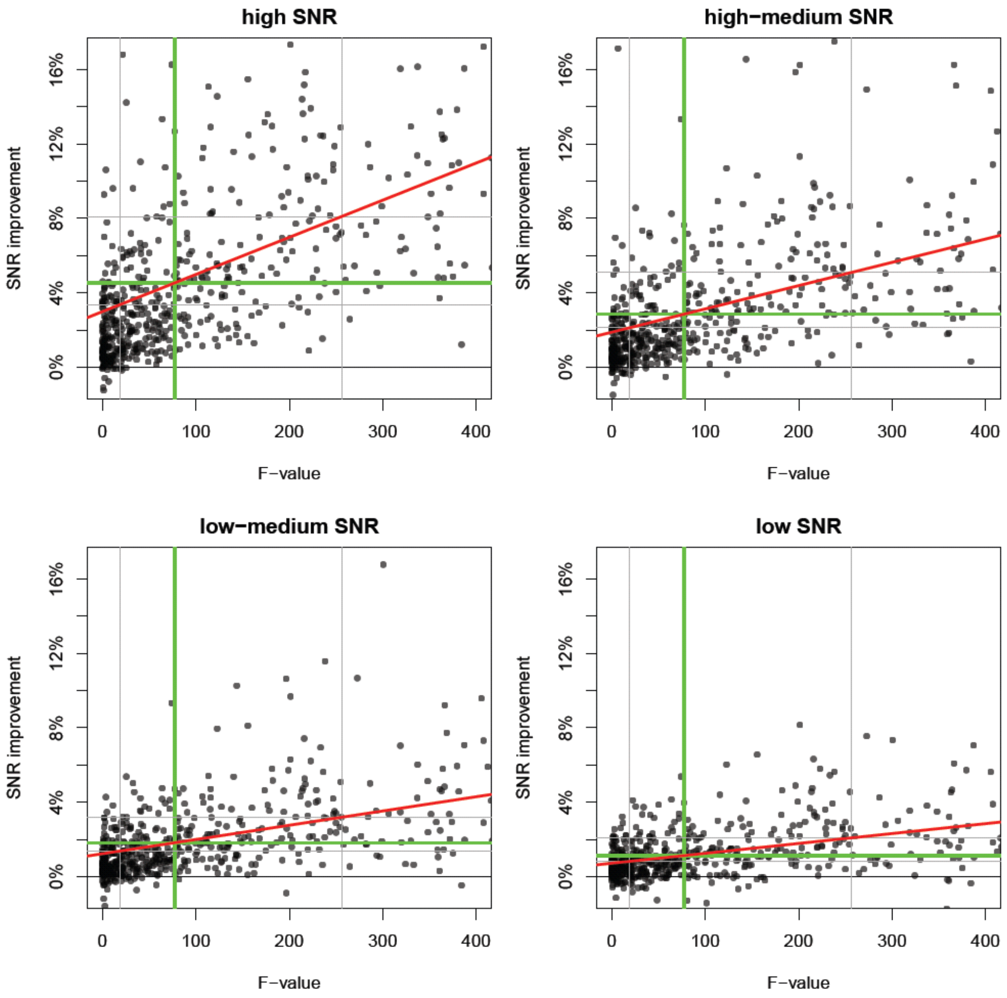

Additional file 1: Figure S9. Improvement by *seqnorm* in comparison to standard regression, measured in SNR increase of the normalized profiles (Y-axis). The improvement is dependent on SNR of the original profile (high, high-medium, low-medium and low), and the F-value (X-axis) of the correlation between GC content and copy number. The red line linearly regresses over the improvements on each SNR level. The green thick lines indicate the median SNR improvement and F-value, while the grey thin lines indicate the corresponding 25% and 75% quantiles. Data is not shown completely for clarity; top SNR improvement ranges from 27% in profiles with low original SNR to 77% in profiles with high original.

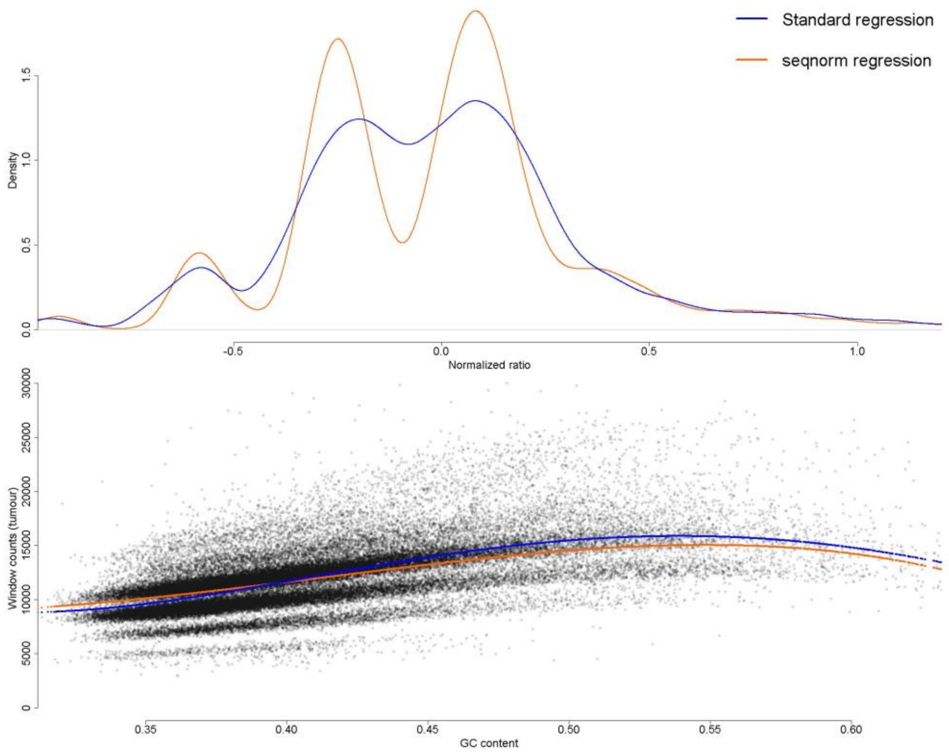

Additional file 1: Figure S10. Normalization of one of the 676 semi-simulated samples. The copy number profile corresponds to the C2BBe1 cell-line sample. The RC variability is based on the matched normal of sample 4076 (4077). The GC content bias was constructed to resemble that of the COLO-829BL sample. Above, density plot of normalized RC profile. Below, correlation between GC and RC that was corrected. In orange, the density and regression line estimated by *seqnorm*. In blue, those by the typical regression.

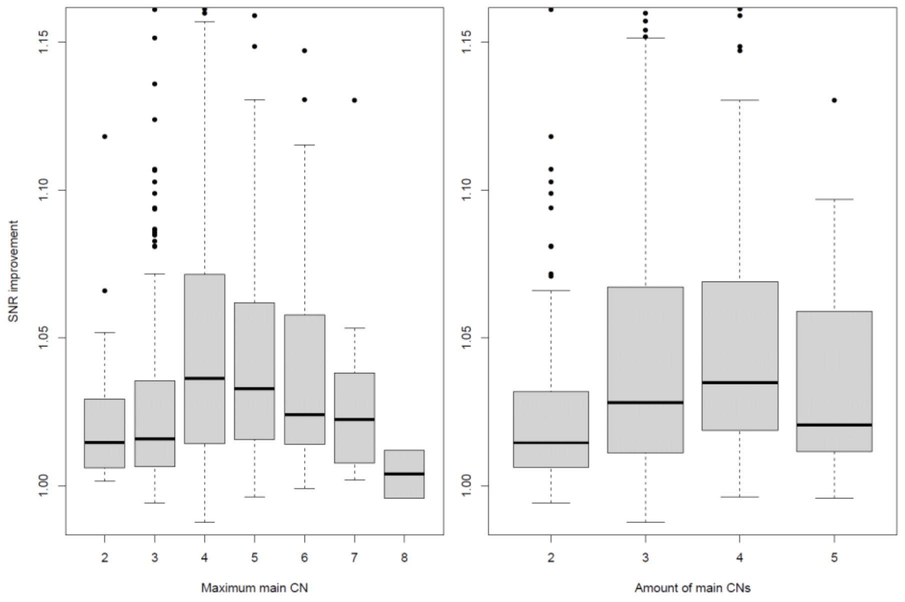

Additional file 1: Figure S11. Boxplots depicting how copy number distribution within samples influences SNR improvement achieved by *seqnorm* with respect to typical regression, under a medium-high original SNR. Left: improvement depending on maximum copy number spanning at least 5% of the genome. Right: improvement depending on the amount of copy numbers spanning at least 5% of the genome.

4.2 RESULTS ON REAL SAMPLES

The clearer profiles obtained with *seqnorm* for the HCC1143 cancer cell-line (Chiang *et al.*, 2008) (Figure 2) and the tumoural sample PR-3027 (Berger *et al.*, 2011) (Additional file 1: Figure S12) further exemplify, on real data, the benefits offered over typical regression in those cases where a correlation between copy number and GC content exists.

These samples present either low coverage (HCC1143) or normal cell contamination (PR-3027), so their profiles have an average SNR even at the low 50Kbps resolution used for their normalization. Given that the improvements with *seqnorm* are greater on samples with high SNR, we expect the introduction of *seqnorm* to be of more relevance as technologies allow higher coverage at lower costs and tumoural cells can be better and more easily separated from normal cells.

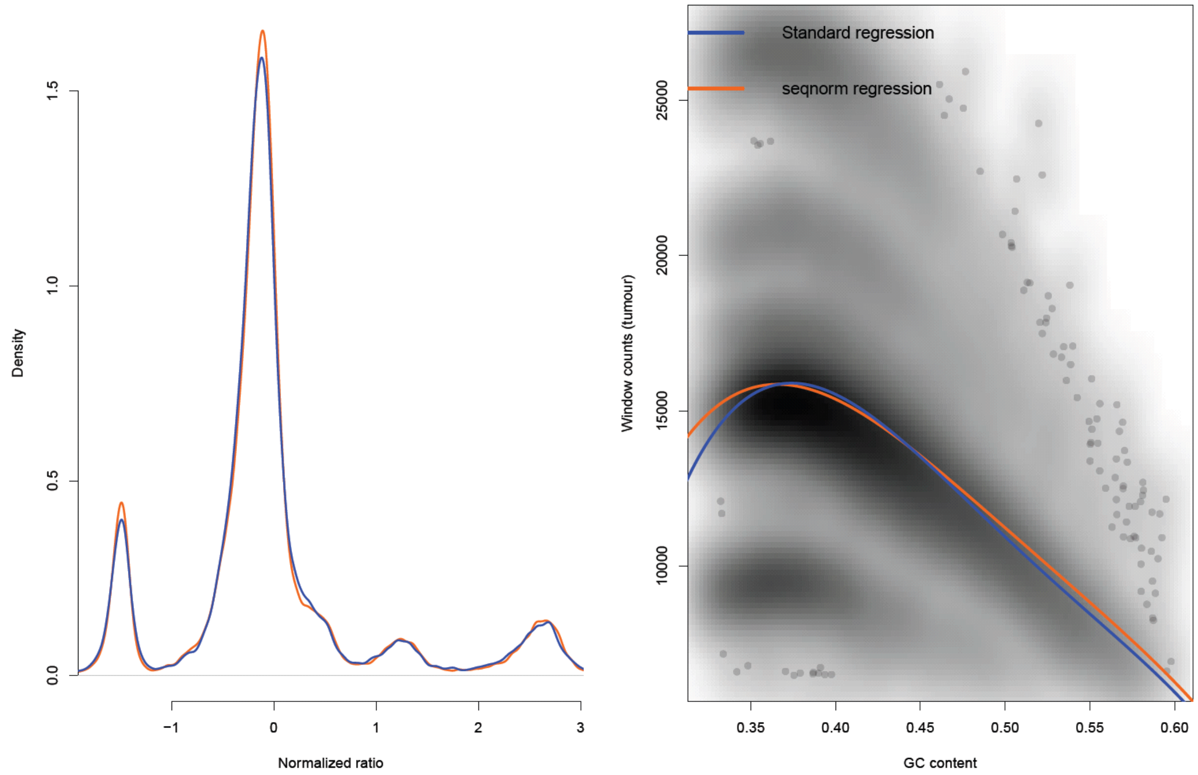

Additional file 1: Figure S12. Normalization results over the PR-3027 tumoural sample's RC profile. (Left) Density plot of the normalized RC profiles. The density of the *seqnorm*-normalized profile distinguishes better the different copy numbers in the mixture. (Right) Read count density depending on the window's GC content, with greater density as darker grey. In blue, regression line estimated through the typical approach. In orange, regression line estimated by *seqnorm*.

5. REFERENCES

Bengtsson,H. *et al.* (2010) TumorBoost: Normalization of allele-specific tumor copy numbers from a single pair of tumor-normal genotyping microarrays. *BMC Bioinformatics*, **11**, 245.

Berger,M.F. *et al.* (2011) The genomic complexity of primary human prostate cancer. *Nature*, **470**, 214–20.

Bignell,G. *et al.* (2010) Signatures of mutation and selection in the cancer genome. *Nature*, **463**, 893–898.

Boeva,V. *et al.* (2011) Genome analysis Control-free calling of copy number alterations in deep-sequencing data using GC-content normalization. *Bioinformatics (Oxford, England)*, **27**, 268–269.

Calenge,C. (2006) The package “adehabitat” for the R software: A tool for the analysis of space and habitat use by animals. *Ecological Modelling*, **197**, 516–519.

Campbell,P.J. *et al.* (2008) Identification of somatically acquired rearrangements in cancer using genome-wide massively parallel paired-end sequencing. *Nature genetics*, **40**, 722–9.

Chiang,D.D.Y. *et al.* (2008) High-resolution mapping of copy-number alterations with massively parallel sequencing. *Nature methods*, **6**, 99–103.

Greenman,C.D. *et al.* (2010) PICNIC: an algorithm to predict absolute allelic copy number variation with microarray cancer data. *Biostatistics (Oxford, England)*, **11**, 164–75.

Gusnanto,A. *et al.* (2012) Correcting for cancer genome size and tumour cell content enables better estimation of copy number alterations from next-generation sequence data. *Bioinformatics (Oxford, England)*, **28**, 40–7.

Langmead,B. *et al.* (2009) Ultrafast and memory-efficient alignment of short DNA sequences to the human genome. *Genome biology*, **10**, R25.

Li,H. *et al.* (2008) Mapping short DNA sequencing reads and calling variants using mapping quality scores. *Genome research*, **18**, 1851–8.

Li,H. and Durbin,R. (2009) Fast and accurate short read alignment with Burrows-Wheeler transform. *Bioinformatics (Oxford, England)*, **25**, 1754–60.

Mayrhofer,M. *et al.* (2013) Patchwork: allele-specific copy number analysis of whole-genome sequenced tumor tissue. *Genome biology*, **14**, R24.

Parisi,F. *et al.* (2011) Detecting copy number status and uncovering subclonal markers in heterogeneous tumor biopsies. *BMC Genomics*, **12**, 230.

Pleasance,E.D. *et al.* (2010) A comprehensive catalogue of somatic mutations from a human cancer genome. *Nature*, **463**, 191–6.

Popova,T. *et al.* (2009) Genome Alteration Print (GAP): a tool to visualize and mine complex cancer genomic profiles obtained by SNP arrays. *Genome Biology*, **10**, R128.

Wang,K. *et al.* (2007) PennCNV: an integrated hidden Markov model designed for high-resolution copy number variation detection in whole-genome SNP genotyping data. *Genome research*, **17**, 1665–74.
